# Relationship between the impacted mandibular third molar and adjacent second molar' external root resorption by cone-bean computed tomography analysis

**DOI:** 10.4317/medoral.26044

**Published:** 2023-11-22

**Authors:** Lian-Yan Cui, En-Shi Jiang, Zhen-Long Liu, Jing-Xu Li

**Affiliations:** 1 DDS. Department of Stomatology, Affiliated Hospital of Yanbian University; 2 DDS, Ph.D. Department of Stomatology, Affiliated Hospital of Yanbian University

## Abstract

**Background:**

The relationship between the impacted mandibular third molar (IMTM) and the external root resorption (ERR) of the mandibular second molar (MSM) was analysed with cone-beam computed tomography (CBCT). The risk factors affecting the ERR of the MSM were examined to provide a reference.

**Material and Methods:**

A total of 327 patients (total: 578 teeth) admitted to the Affiliated Hospital of Yanbian University for IMTM extraction from January 2017 to December 2019 was chosen and divided according to gender and age. The correlation between the IMTM and ERR of MSM was analysed, including inclination angle, impaction direction and depth. The relationship of mandibular ascending ramus classification with ERR of MSM was also analysed. In addition, the correlation between the MTM impaction type and the severity of ERR was analysed.

**Results:**

The incidence of ERR of MSM in male patients was higher than in females (27.9% vs.17.6%, *p* = 0.018). The occurrence and the site of ERR showed statistical differences in the inclination angle [(≤20°, 3.6%) vs. (21°-40°, 27.1%) vs. (41°-60°, 27.6%) vs. (61°-80°, 25.6%) vs. (>80°, 31.7%), *p* <0.001], impaction direction [(Vertical, 1.1%) vs. (Mesial, 32.7%) vs. (Horizontal, 25.3%), *p* <0.001] and depth of MTM [(Low position, 38.6%) vs. (Median position, 32.0%) vs. (High position, 13.7%), *p* <0.001]. Also, there was a significant difference in the mandibular ascending ramus type [(Class I, 17.4%) vs. (Class II, 32.3%) vs. (Class III, 44.9%), *p* <0.001]. In addition, the severity of ERR showed statistical differences in the mesial (40.9%, *p*<0.05), lower impaction (54.5%, *p*<0.05) depth of MTM and type III of mandibular ascending ramus (63.6%, *p*<0.05).

**Conclusions:**

The inclination angle, impaction direction, and depth of MTM were the influencing factors for the occurrence and site of ERR. Also, mandibular ascending ramus type was the impact fact. For MTM with mesioangular, lower impaction, and mandibular ascending ramus with type III, the ERR of the MSM was severer.

** Key words:**Mandibular second molar’s external root resorption, impacted mandibular third molar, cone-bean computed tomograph.

## Introduction

The third molars are the most impacted teeth, and they usually erupt between the ages of 17 and 21 if they are not under impaction (1). The impacted mandibular third molar (IMTM) might cause pericoronitis, caries, odontogenic cysts, tumours, damage to adjacent teeth, anterior teeth crowding, periodontal problems with adjacent second molars, or external root resorption (ERR) (2,3).

The occurrence of ERR goes through two stages: chemical or mechanical injury and stimulation from infection or pressure on protective tissues on the external root surface (4). The ERR of the mandibular second premolar is caused by the second molar’s contact with the mandibular third molar (MTM), indicating that pressure applied by the MTM might cause the ERR of the mandibular second molar (MSM). Such pressure can cause inflammation and the resorption of the MSM through osteoclast action (5). Studies show that ERR is pathological resorption, chiefly occurring on the surface of the root. It causes irreversible damage: mild ERR can cause a shortened or blunt root, and severe ERR can cause dental pulp diseases and other lesions, affecting the stability and chewing functions of the teeth and finally leading to lesioned teeth extraction. Even mild or moderate ERR can cause a decrease in periodontal tissues of the adjacent third molar. However, studies have shown no symptoms when mild or moderate ERR lesions have not reached the dental pulp, so it typically cannot be identified through clinical examination (6), and imaging techniques are required for diagnosis. Therefore, the timely diagnosis of ERR is very important and should be performed as early as possible.

In this study, the relationship between the impacted mandibular third molar (IMTM) and the external root resorption (ERR) of the mandibular second molar (MSM) was analysed with cone-beam computed tomography (CBCT). In addition, the risk factors causing the ERR of the MSM were analysed . The risk factors affecting the ERR of the MSM were examined to provide a reference.

## Material and Methods

- Clinical data

The clinical data were collected from a total of 327 patients (166 males and 161 females, with a total of 578 teeth) admitted to Affiliated Hospital of Yanbian University for the extraction of impacted third molars from January 2017 to December 2019. The patients were aged 18-63 (average: 28.67 ± 9.23). A CBCT examination was further performed on each patient.

The inclusion criteria were as follows: 1) those with complete case data and 2) those with pre-operation CBCT data, fully showing the location relationship between the MTM at the study site and the second molar.

The exclusion criteria were as follows: i. those with MTM or MSM-related cystic lesions, widely distributed caries lesions or abnormalities of the MTM or MSM, third molar development of less than two-thirds, or where the local anatomy and structure of the teeth were covered up due to the existence of high-density materials or other reasons (7); ii. those with diseases related to the MSM, such as dental wounds, chronic periodontitis, or root canal therapy.

- Observation of the CBCT images

Digital images were taken with CBCT (KaVo 3D eXam, Germany), and the images were measured and analyzed with SIMPLANT Pro 17.01 software.

- Determination of the severity of the MSM ERR

The diagnostic criterion was a damaged and irregular root contour with an irregular worm-eaten-like hypodense shadow. The ERR was classified into four types according to severity based on the Ericson classification: non-resorptive, mild, moderate, and severe resorptive (8).

**Figure 1:**
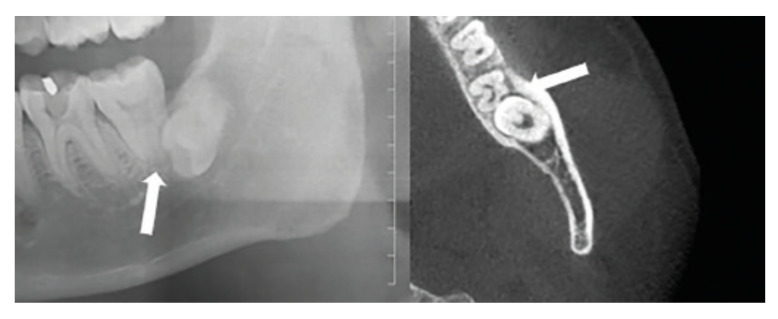
Root Resorption of Mandibular Second Molar (white arrow).

The detailed classification of each class is as follows: i. non-resorptive ERR: a complete root surface that may be accompanied by resorption of the cementum, ii. mild resorptive ERR: root resorption that is not more than half of the root canal wall thickness, iii. moderate resorptive ERR: root resorption that is more than half of the root canal wall thickness but that does not affect the root canal system, and iv. severe resorptive ERR: resorption that affects the root canal system (Fig. 1).

- MTM impaction direction

According to Winter’s classification, IMTM includes the following types based on the angle formed between the long axis of the MSM and the MTM: vertical impaction is -10° to 10°, mesial impaction is 11° to 79°, horizontal impaction is 80° to 100°, distal impaction is -11° to -79°, inverted impaction is 111° to -80°, and buccolingual impaction (9).

- MTM impaction depth

According to Pell and Gregory’s classification, the impaction depth is classified into the following groups based on the depth of the MTM: i. high position: the apex of the crown is flush with or above the occlusal plane, ii. median position: the apex of the crown is below the occlusal plane but above the cervical margin of the second molar, and iii. low position: the apex of the crown is below the cervical margin of the second molar.

- MTM tilt angle

The MTM tilt angle was measured on the sagittal plane with the angle measuring function of the SIMPLANT Pro 17.01 software. According to the methods of Shiller (10), the tilt angle was obtained by measuring the implant angle of the intersection between the MTM occlusal plane and the second molar occlusal plane. The mean value was obtained from the values of three measurements to an accuracy of 1°. The tilt angle was classified into the following groups: ≤20°, 21°-40°, 41°-60°, 61°-80°, and >80°.

- Relationship between the MTM, mandibular ascending ramus, and second molar

The ascending ramus was classified into types I, II, and III according to the relationship between the MSM and MTM and based upon Pell and Gregory’s classification. These classes are defined as follows: i. type I: there is space between the front edge of the mandibular ascending ramus and the distal plane of the second molar, which is sufficient to contain the mesio-distal dimension of the third molar crown; ii. type II: the distance between the mandibular ascending ramus and the second molar is less than the mesio-distal dimension of the third molar crown; and iii. type III: the whole or majority of the MTM is within the mandibular ramus.

- Division into thirds of the MSM root

The tooth was divided into three equal parts according to the root resorption: one-third root cervical, one-third root medial, and one-third root tip.

- Statistical analysis

Statistical analysis was conducted using SPSS 26.0 software. Enumeration data were checked using the Chi-square test, and between-group comparisons were performed using Bonferroni’s test. Factors with statistical significance in the univariate analysis of the MSM ERR were included in a multi-factor bivariate logistic regression analysis. A value of *p* < 0.05 was deemed statistically significant.

## Results

- Existence of second molar ERR and its relationship with clinical characteristics

Among 327 CBCT images, ERR was identified in the distal plane of 138 second molars among 578 teeth, with a 23.9% prevalence. The incidence of ERR showed statistical differences in respect of gender (*p* < 0.05): the incidence in male patients was 27.9%, and in female patients, it was 19.6%. The incidence of ERR of the second molar showed no statistical differences in respect of age (*p* > 0.05) (Table 1).

The incidence of ERR showed statistical differences in respect of different MTM impaction directions (*p* < 0.05): for vertical, mesial, and horizontal impaction, the incidence of second molar ERR was 1.1%, 32.7%, and 25.3%, respectively. The incidence of ERR showed statistical differences in respect of MTM tilt angles (*p* < 0.05): when the MTM tilt angle was ≤20°, 21°-40°, 41°-60°, 61°-80°, or >80°, the incidence of second molar ERR was 3.6%, 27.1%, 27.6%, 25.6%, or 31.7%, respectively. The incidence of ERR showed statistical differences in respect of different MTM impaction depths (*p* < 0.05): when the MTM impaction was low, medium, or high, the incidence of the second molar ERR was 38.6%, 32.0%, or 13.7%, respectively. The incidence of ERR showed statistical differences in respect of the ascending ramus type (*p* < 0.05): when the MTM was type I, II, or III, the incidence of second molar ERR was 17.4%, 32.3%, or 44.9%, respectively (Table 1).

- Multi-variable logistic regression analysis on the influencing factors of the MSM ERR

Factors with statistical significance in the univariate analysis were included in the bivariate logical regression model. Bivariate logical regression analysis was conducted with gender, impaction direction, tilt angle, impaction depth, and ascending ramus type as concomitant variables. As seen in the results, the incidence of ERR in respect of gender showed no statistical significance (*p* > 0.05). The incidence of horizontal and mesial impaction ERR was 41.951 (*p* < 0.05) and 76.212 (*p* < 0.05) times that of vertical impaction, respectively. The incidence of mid and low-position impaction ERR was 2.097 (*p* < 0.05) and 2.988 (*p* < 0.05) times that of high-position impaction, respectively. The incidence of types II and III ERR was 1.743 (*p* < 0.05) and 2.344 (*p* < 0.05) times that of type I, respectively. The incidence of ERR in respect of the tilt angle showed no statistical differences (*p* > 0.05) (Table 2).

- Relationship between the severity of second molar ERR and clinical characteristics

Further statistical analysis was performed on the factors affecting the severity of second molar ERR, and the results showed no statistical differences in respect of age, impaction direction, and resorption site (*p* > 0.05).

**Table 1:**
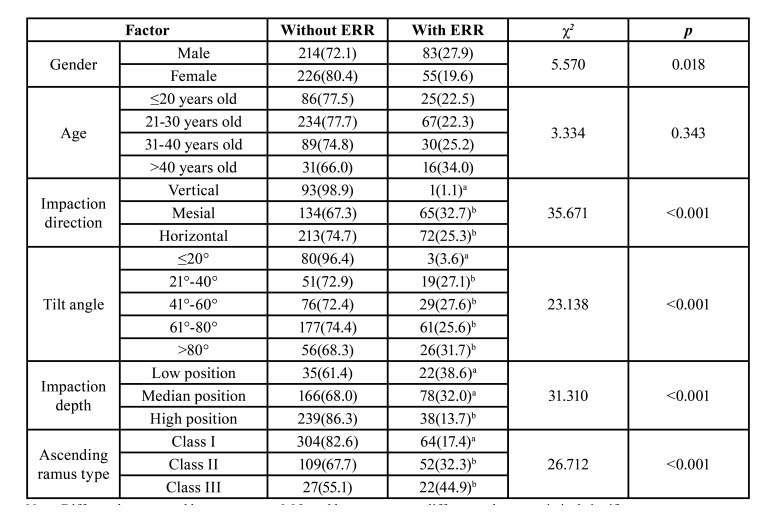
Analysis on ERR of Second Molar [n(%)].

**Table 2:**
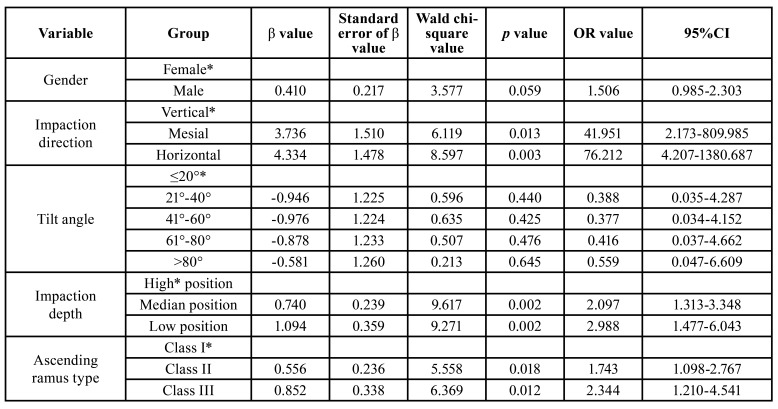
Multi-variable logistic regression analysis on influencing factors of ERR of mandibular second molar.

However, the results showed statistical differences in respect of different MTM impaction depths (*p* < 0.05) as follows: i. a high incidence of second molar moderate resorption upon low-position MTM impaction (40.9%), ii. a high incidence of second molar mild resorption upon median-position MTM impaction (78.2%), and iii. a high incidence of second molar mild resorption upon high-position MTM impaction (60.5%) (Table 3).Relationship between different ERR sites and MTM impaction types.

The MTM impaction depths showed statistical differences in respect of second molar ERR sites (*p* < 0.05), and the most common resorption site was the root tip third for low-position impaction (54.5%). The most common resorption site was the root medial third for median-position impaction (59.0%), and the most common resorption site was the root cervical third for high-position impaction (89.5%). The MTM impaction direction showed statistical differences in respect of second molar ERR sites (*p* < 0.05), and the most common resorption site was the root cervical third for mesial impaction (73.8%). The most common resorption site was the root medial third for horizontal impaction (52.8%). The MTM tilt angle showed statistical differences in respect of second molar resorption sites (*p* < 0.05). When the tilt angle was ≤20°, the most common resorption site of the second molar was the root cervical third (66.7%). When the tilt angle was 21°-40°, the most common resorption site of the second molar was the root cervical third (78.9%). When the tilt angle was 41°-60°, the most common resorption site of the second molar was the root cervical third (75.9%), and when the tilt angle was 61°-80°, the most common resorption angle of the second molar was the root medial third (47.5%). Moreover, when the tilt angle was >80, the most common resorption site of the second molar was the root medial third (57.7%). The ascending ramus type showed statistical differences in respect of second molar ERR sites (*p* < 0.05). The most common resorption site for type I was the root cervical third (54.7%), and the most common resorption site for type II and III was the root medial third, covering 46.2% and 63.6%, respectively. (Table 4).

**Table 3:**
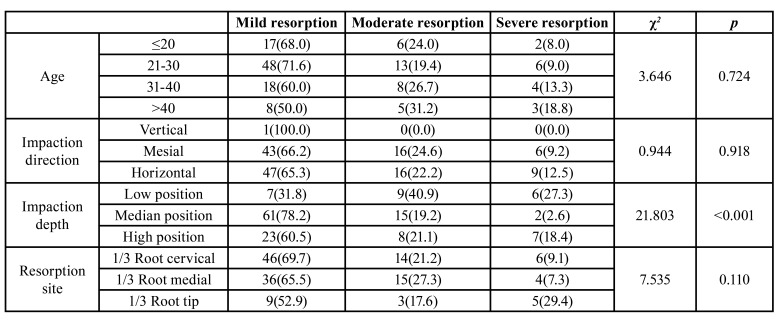
Relationship between ERR Severity of Second Molar and Clinical Characteristics [n(%)].

**Table 4:**
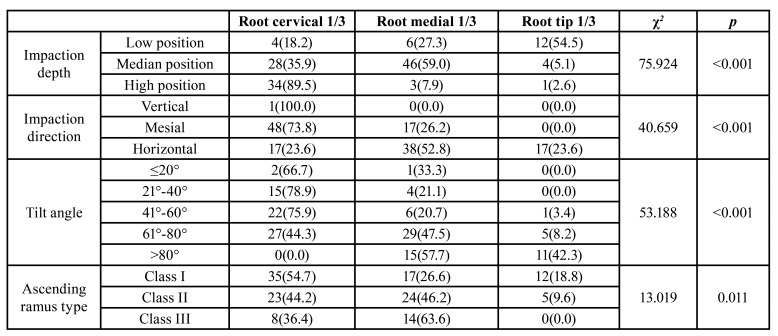
Relationship between Different ERR Sites and MTM Impaction Types [n(%)].

Discussions

This study aims to investigate the MTM impaction types and the risk factors of second molar ERR. Impacted teeth might be associated with some pathological conditions: pericoronitis, caries, bone defects, odontogenic cysts, tumors, and adjacent teeth ERR (11,12-14), and ERRs are rarely seen. When resorption affects the dental pulp, causing second molar inflammation, extraction of the impacted third molar can eliminate mechanical stress of the distal plane of the second molar to slow down the development of inflammation. When bacteria invade the dental pulp, root canal therapy is required for the second molar. According to recent studies, upon the impaction of the third molar, the crown of more than 90% of MTMs comes into contact with the adjacent second molar, so this might be the cause of second molar ERR (15).

Nemcovsky *et al*. (16) conducted clinical and histological examinations for second molars with ERR. In their study, ERR was found in all histological samples, but not all second molars were detected with ERR in clinical examinations (17). The clinical and histological examinations might not be available for all cases, but 3D high-resolution images of CBCT can accurately identify such pathological conditions. In previous studies, the prevalence of second molar ERR near-impacted MTM was detected as 0.3-24.2% with different imaging techniques (5,16). However, due to excessive projection of the anatomic structure, it was difficult to evaluate the existence of ERR on panoramic radiographs. In a retrospective study with a panoramic radiograph, nearly half of the cases were observed with an overlapping of the second and third molars (5). Oenning *et al*. (18) detected an even higher prevalence of second molar ERR with CBCT (49.43%). In the present study, the incidence of second molar ERR in all patients was 23.9% as observed by CBCT imaging, which is consistent with the results of previous research (16). However, compared with the CBCT clinical imaging of Oenning *et al*. (18), the prevalence of ERR in this study was lower. It is deduced that such differences may be associated with different sample sizes, patient selection bias, and different inclusion criteria. Therefore, a larger number of patients from different hospitals should be included according to the same inclusion criteria to further determine the prevalence of second molar ERR related to impacted MTM. The results of this study are consistent with the results of Wang *et al*. (7) using CBCT (20.17%). Some studies that demonstrated the occurrence of ERR were not associated with gender (19), but the incidence of second molar ERR showed statistical differences in respect of gender (*P* < 0.05) in the current study. The incidence of ERR in male patients was higher than that of female patients (27.9%), which is consistent with the study results of Yamaoka *et al*. (5). In other studies, it was found that the incidence of ERR in male patients as analyzed with CBCT was two times that of female patients (20), possibly due to the interference of hormones in the identification of a higher incidence of second molar ERR in male patients. Estrogen in females can prevent bone resorption, so the risk of ERR in females is lower (21). Therefore, the incidence of ERR in male patients was higher than that in female patients.

One of the risk factors of ERR analyzed in this study is the age of the patients. After the formation of the dental root, the eruption movement of the teeth continues, and the teeth continually apply mechanical pressure to adjacent second molars, providing conditions for ERR progression (22). It was found in early studies with periapical film and CBCT that ERR was positively related to age (7,23). Previous study results suggest a higher incidence of ERR in patients aged 24 or above (11), and Wang *et al*. (7) found an age of 35> to be an independent risk factor for ERR. The results of the current study show that second molar ERR was not associated with age, which is consistent with the study of Ericson *et al*. (24) The results of studies on ERR caused by pressure also reveal no association between ERR and the age of patients because severe resorption of pulp exposure was observed at the early stage of canine teeth eruption.

The present study demonstrates a higher incidence of ERR in teeth adjacent to third molars with a medial (32.7%) and horizontal impaction (25.3%), which is consistent with the study of Smailien *et al*. (15). Matzen *et al*. (25) also found that ERRs were mostly associated with the third molar having a medial or horizontal impaction, as there was a larger contact area between horizontally impacted third and second molars, providing opportunities for higher resorptive pressure. Oenning *et al*. (26) determined, by finite element analysis, that stress centralized on the contact site was caused by close contact between the MTM and adjacent teeth. With horizontal impaction of the MTM, the main stress and deformation site occurred between the second and third molars. To support this opinion, it is worth noting that the MTM tilt angle was quantified in the study to reduce observation deviation, and it was found that the risk of MTM ERR was higher when the tilt angle was >80°. This is different from the results of previous studies. Some studies showed that the risk of ERR was higher when the angle between the second and third molar was 44.07°-68.01° (27). Li *et al*. (28) found that when the MTM tilt angle was 46°-75°, the risk of ERR was higher. This was possibly due to the larger contact area between the third and second molars at this angle. This also indicated that when MTM stress was located at a certain area on the distal surface of the second molar, there was a high probability of second molar ERR. Therefore, patients with a >80° angle should be informed of the risk of ERR by the dentist, even if no ERR resorption was identified in the examination. To determine the angle range between the MTM and the second molar and the risk of ERR in more detail, the angle measurement should be performed with CBCT in future studies. Therefore, for cases of mesial impaction and a >80° tilt angle, the incidence of ERR was higher.

In the present study, it was found that vertically impacted third molars also caused second molar ERR. The reason might be the higher eruption force after the partial eruption of the impacted teeth, which causes damage to the root cementum and second molar dentin under osteoclastic action, leading to ERR. Wang *et al*. (7) demonstrated that, compared with mid-position impaction, the risk of high and low positions causing mandibular second molar ERR was higher. This is consistent with the results in this study that indicate that the high prevalence of second molar ERR is caused by low-position impaction. Another factor that causes ERR might be the available eruption space for the MTM, but some studies have shown no significant correlation between the occurrence of ERR and the eruption space of the MTM (15). It was found in this study, however, that the occurrence of ERR was associated with the eruption space of the MTM, which is different from the results of previous studies. Type III MTM was more prone to causing second molar ERR, which occurred at the root medial third. This indicates that the contact area between the third molar crown and the second molar root became larger, and a certain pressure was caused when the majority or whole of the third molar was in the mandibular ascending ramus, leading to ERR. According to the logistic multivariate regression analysis in this study, the risk of mesial impaction causing ERR was 76.212 times that of vertical impaction. The risk of low-position impaction causing ERR was 2.988 times that of high-position impaction, and the risk of type III causing ERR was 2.344 times that of type I, indicating that mesial impaction, low-position impaction, and type III third molar were the risk factors for ERR.

Some studies demonstrate that the severity of ERR increases with age (28). Such a relationship was not found in this study, possibly due to the different inclusion and exclusion criteria and the selection bias. However, it was apparent that the incidence of mild resorption was higher between the ages of 21-40, and the incidence of severe resorption was lower at age >40. This was possibly due to patients making more hospital visits, leading to the MTM being extracted before the occurrence of symptoms in the second molar. In this study, it was found that the impaction direction was not associated with the severity of ERR, which is consistent with the study results of Smailien *et al*. (15). Studies have shown that mild and moderate resorption is more frequently observed at the root cervical third and that ERR at the root tip third is the most severe (19). This study revealed that the severity of ERR was not significantly correlated with the resorption site of ERR, which is consistent with the results of Wang *et al*. (7). However, it was found in the current study that the impaction depth of the MTM was associated with the severity of second molar ERR, usually with a higher incidence of moderate resorption for low-position impaction and a higher incidence of mild resorption for high-position impaction. This was consistent with the study results of Smailien *et al*. (15). Studies have shown that the severity was associated with the resorption site, with mild resorption occurring at the root cervical and severe resorption at the root tip. The cervical and tip areas might be susceptible to ERR (15). However, the present study suggests that the severity of ERR was not associated with the resorption site, which is different from the results of previous studies, but it could be seen that most of the mild and severe resorption of the second molar ERR occurred at the root cervical third, indicating that the cervical areas were susceptible to ERR.

In this study, the impaction depth was found to be associated with the resorption sites of ERR, which is consistent with the results of Lacerda-Santos *et al*. (19). The site of the second molar ERR caused by the third molar with low-position impaction was mostly at the root tip third, and the site was mostly at the root cervical third for high-position impaction. This may be because the pressure at the dental ligament and on the distal surface of the dental root decreases upon the partial eruption of the third molar, or it may be because the cementum at the root apex is much softer than the cementum of the dental root (29). Some studies have shown that the impaction direction was not associated with the resorption sites of ERR (19), while it was found in the current study that ERR occurred mostly at the root cervical third for mesial impaction of the MTM. This indicates that the root cervical area is susceptible to ERR and that inflammation is more likely to occur in exposed areas, such as the junction area of the enamel and cementum (20). Therefore, the resorption site of ERR was subject to the impaction depth of the MTM, low-position impaction of the MTM easily caused moderate resorption at the root tip third, and mesial impaction easily caused resorption at the root cervical third.

It should be noted that ERR might be caused by multiple pathogenic factors, so the location characteristics of the impacted third molar might not be the sole risk factor for ERR. The hereditary inclination of ERR, physiological features of reaction of hard tissues toward pressure, and the inflammation process may also be associated with ERR (30). Compared with previous similar studies, a larger sample size was chosen for this study, providing more adequate arguments for the conclusion. No cases were included for second molar ERR caused by buccolingual or inverted third molar because only the inclination of the third molar at the sagittal direction can cause second molar ERR, but this may have caused sample selection bias.

There are some limitations to this study. For example, patients, after undergoing strict screening according to the inclusion criteria, could not represent the average sample for the MTM, which caused sample selection bias. Furthermore, few cases of MTM with vertical impaction and distal impaction were included in this study, so the sample size should be expanded and more research centers should be included for further studies in the future.

## Conclusions

The impaction direction, impaction depth, and ascending ramus type were the influencing factors for second molar ERR. The impaction direction, tilt angle, impaction depth, and ramus type affected the site of ERR. For MTM with mesioangular impaction, lower impaction, and type III, the incidence of second molar ERR was higher.
